# Expanding agroinfiltration host range with broad-spectrum effectors monitored by autoluminescent reporter

**DOI:** 10.1093/hr/uhag126

**Published:** 2026-04-06

**Authors:** Tiange Wang, Jieyu Ge, Chengyi Qu, Zhenkai Wang, Hongyu Chen, Jie Li, Shun Wang, Xueying Guan, Tao Chen, Zhanfeng Si, Keming Hu, Xinhua Ding, Li Liu, Jian Zhang, Alexander S Mishin, Ilia V Yampolsky, Hao Du

**Affiliations:** College of Agriculture and Biotechnology, Zhejiang University, Hangzhou 310058, China; College of Agriculture and Biotechnology, Zhejiang University, Hangzhou 310058, China; ZJU-Hangzhou Global Scientific and Technological Innovation Center, Zhejiang University, Hangzhou 311215, China; College of Agriculture and Biotechnology, Zhejiang University, Hangzhou 310058, China; College of Agriculture and Biotechnology, Zhejiang University, Hangzhou 310058, China; College of Agriculture and Biotechnology, Zhejiang University, Hangzhou 310058, China; Hebei International Research Centre of Vegetable Functional Genomics, College of Horticulture, Hebei Agricultural University, Baoding 071000, China; College of Agriculture and Biotechnology, Zhejiang University, Hangzhou 310058, China; Yazhouwan National Laboratory, Sanya 572024, China; College of Agriculture and Biotechnology, Zhejiang University, Hangzhou 310058, China; College of Life and Environmental Science, Hangzhou Normal University, Hangzhou 311121, China; College of Agriculture and Biotechnology, Zhejiang University, Hangzhou 310058, China; Jiangsu Key Laboratory of Crop Genomics and Molecular Breeding/Key Laboratory of Plant Functional Genomics of the Ministry of Education, College of Agriculture, Yangzhou University, Yangzhou 225009, China; State Key Laboratory of Crop Biology, Shandong Provincial Key Laboratory for Biology of Vegetable Diseases and Insect Pests, College of Plant Protection, Shandong Agricultural University, Taian 271018, China; State Key Laboratory of Biocatalysis and Enzyme Engineering, Hubei Collaborative Innovation Center for Green Transformation of Bio-Resources, School of Life Sciences, Hubei University, Wuhan 430062, China; State Key Laboratory of Rice Biology and Breeding, China National Rice Research Institute, Hangzhou 311400, China; Shemyakin-Ovchinnikov Institute of Bioorganic Chemistry, Russian Academy of Sciences, Moscow 117997, Russia; Shemyakin-Ovchinnikov Institute of Bioorganic Chemistry, Russian Academy of Sciences, Moscow 117997, Russia; College of Agriculture and Biotechnology, Zhejiang University, Hangzhou 310058, China; ZJU-Hangzhou Global Scientific and Technological Innovation Center, Zhejiang University, Hangzhou 311215, China

## Abstract

*Agrobacterium*-mediated transient transformation is a fundamental tool for plant research and molecular pharming, but its application is often limited by host immune responses, post-transcriptional gene silencing, and the lack of a robust, quantitative reporter for species beyond *Nicotiana benthamiana*. To address these challenges, we developed a sensitive reporter system based on the fungal bioluminescence pathway (FBP). As an autonomous, substrate-free metabolic pathway, the FBP enables high-throughput, quantitative screening without external manipulations. Using this system, we systematically evaluated factors affecting transformation efficiency across more than 20 plant families. This cross-lineage analysis identified that co-expressing NahG and P19, termed the NaP19 module, not only mitigates P19-induced cytotoxicity but also synergistically enhances transient expression in diverse species, including agroinfiltration-recalcitrant crops and horticultural plants. We demonstrate the platform's *in vivo* utility in its native genetic background for applications, such as protein localization, interaction assays, and transcriptional regulation studies. By integrating a quantitative, substrate-free reporter with a broadly effective NaP19 enhancer, this work establishes a robust and versatile platform for advancing functional genomics and biotechnology throughout the land plant lineages.

## Introduction

Transient expression in plants represents a powerful and rapid approach for high-throughput analysis of gene function, significantly accelerating research in functional genomics. Among the established systems, transient expression in plant mesophyll protoplasts has served as a versatile platform for cell-based studies, enabling molecular, biochemical, cellular, and proteomic investigations into diverse signaling pathways and cellular mechanisms [1, 2]. Nevertheless, the isolation of high-quality protoplasts capable of supporting efficient transgene expression remains technically challenging, time-consuming, and costly. This limitation is especially pronounced in non-model plant species, where robust protocols for protoplast isolation and transformation are often underdeveloped. As an alternative, agroinfiltration of *Nicotiana benthamiana* has emerged as a widely adopted system for rapid production of recombinant proteins, demonstrating considerable utility in both molecular pharming and basic research [3, 4]. When compared to mammalian cell culture or microbial fermentation systems, agroinfiltration in plants offers distinct advantages, including lower production costs, reduced risk of contamination with human pathogens, high scalability, and the capacity to perform eukaryotic post-translational modifications, such as glycosylation and disulfide bond formation [5, 6].

Despite these benefits, the application of agroinfiltration-based transient expression remains constrained in many plant species including crops, ornamentals, and perennial trees, due to limited efficiency and generalizability. Although alternative techniques, such as biolistic bombardment and *Agrobacterium*-mediated infiltration, have been adapted for transient transformation in certain species, like *Arabidopsis thaliana*, *Solanum tuberosum*, and *Capsicum annuum* [7–9], their broader implementation is often hampered by dependencies on specialized equipment, the high cost of consumables, such as gold microparticles, and persistently low transformation efficiencies. Beyond standard syringe agroinfiltration, alternative platforms, such as viral vectors and vacuum-based methods, have been developed to enhance transient expression. Viral vectors amplify transgene replication and systemic movement but often elicit strong immune responses, limiting their suitability for studying endogenous protein interactions and transcriptional regulation. Vacuum-based agroinfiltration enables large-scale tissue infiltration yet requires specialized equipment and facilitates only bacterial entry without improving infection efficiency or transgene expression [10]. These limitations underscore the need for improved syringe-based agroinfiltration methods that combine enhanced efficiency with broad applicability.

Plant resistance to *Agrobacterium* significantly limits transformation efficiency by activating immune responses upon bacterial detection. Specifically, *Agrobacterium*-derived pathogen-associated molecular patterns (PAMPs) trigger PAMP-triggered immunity (PTI) [11], which restricts transient gene expression. To overcome this barrier, several strategies have been explored. For instance, expression of the bacterial Type III effector AvrPto can suppress immune-related kinases and enhance transformation in certain plant systems [12]. Salicylic acid (SA) is a central regulator of plant defense. Beyond activating defense genes, SA induces RNA-dependent RNA polymerases (RdRP), which amplify RNA silencing signals from exogenous transcripts and promote the silencing of foreign genes [13, 14]. During agroinfiltration, elevated SA levels can trigger these pathways, limiting transient transgene expression. The bacterial salicylate hydroxylase NahG degrades SA, offering a dual benefit: it attenuates SA-mediated defense responses and suppresses SA-induced RdRP activity, thereby enhancing transient expression efficiency [15–17]. However, the broader applicability of this SA-depletion approach across diverse plant species remains unclear.

Beyond innate immunity, post-transcriptional gene silencing (PTGS) represents another major obstacle to sustained transgene expression. PTGS typically initiates several days after infiltration through the production of double-stranded RNAs, which are processed into small interfering RNAs (siRNAs) that guide mRNA degradation [18]. Silencing can be mitigated by genetic disruption of Dicer-like proteins (*e.g. dcl2dcl4* mutants) or through the expression of viral suppressors of RNA silencing (VSRs) [19, 20]. Among VSRs, the tombusviral protein P19 has been extensively characterized and functions by sequestering siRNAs, thereby preventing mRNA degradation [21]. Nevertheless, the efficacy of P19 in enhancing agroinfiltration across a wide range of plant hosts has not been systematically established.

The development of robust reporter systems is essential for evaluating and optimizing protein expression platforms in plants. Widely used reporters, such as green fluorescent protein (GFP), require external illumination and are unsuitable for non-destructive quantitative assays under natural growing conditions [22–24]. While luciferase-based reporters offer high sensitivity and quantitative accuracy, their application is often constrained by the high cost of substrates and the requirement for destructive sampling, which precludes longitudinal monitoring of the same living tissues over time [25, 26]. Alternative systems, including β-glucuronidase (GUS) and animal-derived luciferase, rely on costly substrates and involve destructive sampling, thereby limiting scalability and real-time monitoring [27]. Although the visible pigment-based reporter RUBY enables noninvasive detection [28], its utility is constrained by low sensitivity, poor quantifiability, and high background interference. These limitations highlight the need for a sensitive, quantifiable, and non-destructive reporter system suitable for large-scale and in planta applications.

Bioluminescence systems, particularly the fungal bioluminescence pathway (FBP) from mushrooms, offer a promising alternative. The FBP consists of four core enzymes, HispS, H3H, Luz, and CPH, which convert endogenous caffeic acid, a phenylpropanoid metabolite conserved across land plants, into luciferin, resulting in autonomous light emission without exogenous substrates [29, 30]. This self-sustaining pathway has recently been engineered into plants as an autoluminescent reporter [31–34]. In this study, we establish the FBP as a quantitative, substrate-free reporter and demonstrate its compatibility with the immune-suppressing and mRNA-stabilizing module NaP19. This integrated approach significantly enhances transient expression across diverse species, including agroinfiltration-recalcitrant crops and horticultural plants, providing a versatile and scalable platform for high-throughput *in vivo* gene functional analysis.

## Results

### The FBP-based reporter system is applicable across broad land plant lineages

**Figure 1:**
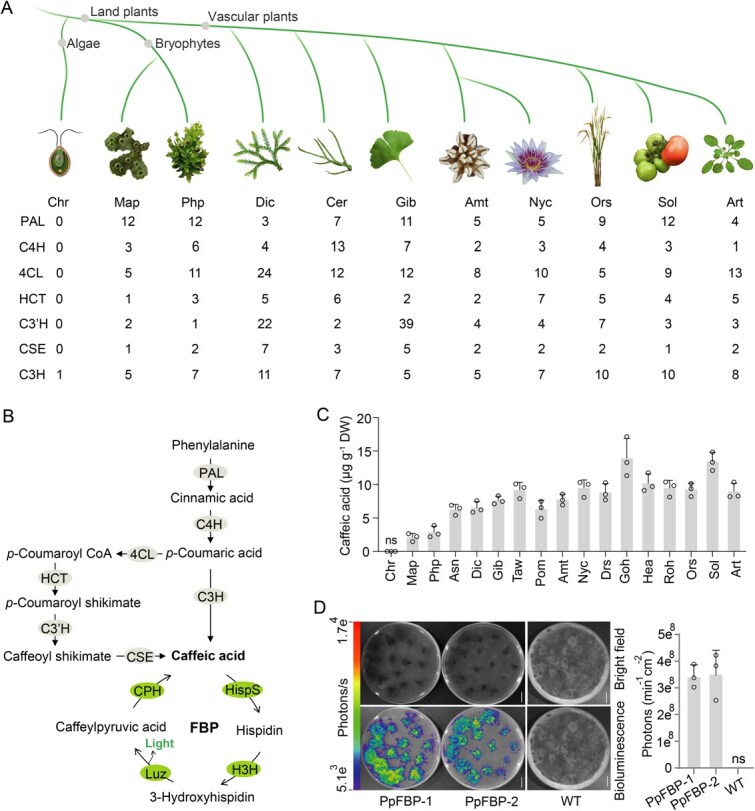
FBP precursor caffeic acid is present in land plant lineages. (A) Phylogenetic tree of the algae and 10 land plant species used in this study. Species marked with gray dots correspond to lateral branching. The number represents the potential number of caffeic acid synthesis related genes in each species. PAL, phenylalanine ammonia-lyase; C4H, cinnamic acid 4-hydroxylase; 4CL, 4-hydroxycinnamate: CoA ligase; HCT, 4-hydroxycinnamoyl CoA: shikimate/quinate hydroxycinnamoyl transferase; C3′H, 4-coumaroyl shikimate/quinate 3′-hydroxylase; CSE, caffeoyl shikimate esterase; C3H, coumarate 3-hydroxylase. Chr, *C. reinhardtii*; Map, *M. polymorpha*; Php, *P. patens*; Dic, *D. complanatum*; Cer, *C. richardii*; Gib, *G. biloba*; Amt, *A. trichopoda*; Nyc, *N. colorata*; Ors, *O. sativa*; Sol, *S. lycopersicum*; Art, *A. thaliana*. (B) Integrated the pathway of plant caffeic acid biosynthesis into the FBP cycle. The black arrows represent direct biochemical reactions, and FBP key genes are highlighted with the green background. (C) LC–MS/MS analysis of caffeic acid content in leaves of different species. Asn, *A. nidus*; Taw, *T. wallichiana*; Drs, *D. sanderiana*; Goh, *G. hirsutum*; Hea, *H. annuus*; Roh, *R. hybrida*. (D) Detection and quantification of autoluminescence in FBP transgenic *P. patens*. Scale bars, 1 cm. Values are mean ± SD (*n* = 3). ns, no signal.

The FBP has recently been reconstituted in plants, enabling substrate-free luminescence imaging and offering a promising alternative to conventional reporter systems [26, 35]. Since the FBP utilizes caffeic acid as its biosynthetic precursor, we hypothesized that its feasibility as a universal plant reporter would depend on the evolutionary conservation of caffeic acid biosynthesis across broad land plant lineages. To test this, we analyzed high-quality genomes from 11 species representing major plant lineages, from algae to angiosperms (Fig. 1A, Table S1). Our results show that land plant lineages retain seven key enzymes (PAL, C4H, 4CL, HCT, C3′H, C3H, and CSE) required for caffeic acid production from phenylalanine (Figs S1 and S2). In contrast, these genes are absent in green algae, such as *Chlamydomonas reinhardtii*, consistent with their inability to synthesize caffeic acid (Fig. 1A and B). These findings indicate that the caffeic acid pathway is widely conserved across land plants, suggesting the broad applicability of the FBP as an autoluminescent reporter in bryophytes and vascular plants alike.

Metabolite analysis across 17 representative plant species confirmed the presence of caffeic acid in both bryophytes and vascular plants, while algae completely lacked this compound, consistent with the evolutionary loss of its biosynthetic genes (Fig. 1A and C). Notably, vascular plants accumulated 2.1- to 4.6-fold higher levels of caffeic acid than bryophytes, reflecting enhanced phenylpropanoid flux [31, 36], consistent with the increased lignin biosynthesis required for structural support. Constitutive expression of the FBP via *Agrobacterium*-mediated transformation produced strong autoluminescence in several vascular plants, including *N. benthamiana*, *Phalaenopsis aphrodite*, and *Chrysanthemum morifolium*, as well as in stable transgenic *Populus canadensis*. In contrast, FBP-transformed *Physcomitrella patens* exhibited autoluminescence approximately two orders of magnitude weaker than that in tobacco [31], correlating with its lower endogenous caffeic acid content (Fig. 1C and D). These results indicate that while the caffeic acid biosynthesis pathway is functionally conserved across land plants, its expression level varies substantially between lineages, directly influencing FBP-driven luminescence intensity. Thus, the FBP system serves as a versatile reporter tool across vascular plants, with precursor availability as a key determinant of performance.

### The NaP19 (NahG–P19) module synergistically enhances *Agrobacterium*-mediated transient expression

**Figure 2:**
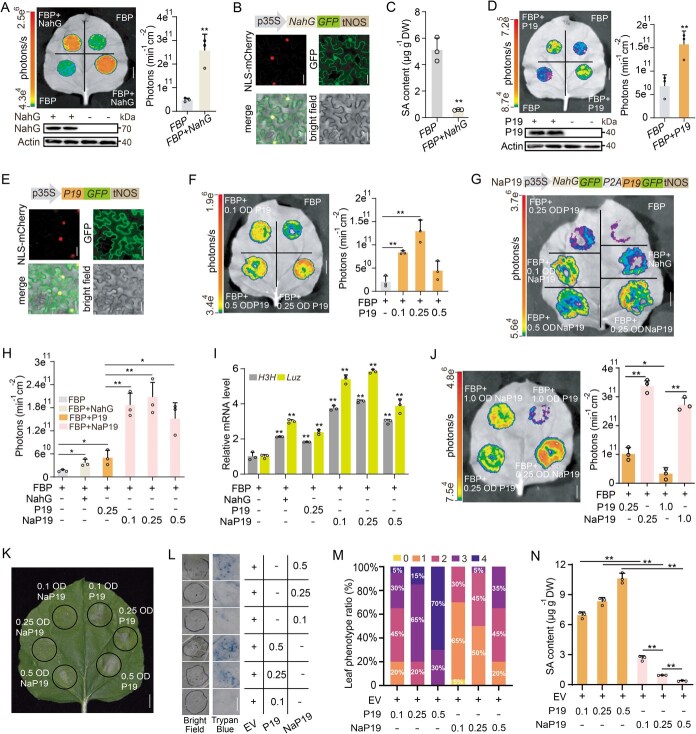
Identification of *NahG* and *P19* effectively enhances agroinfiltration efficiency with the FBP-based reporter system. (A) NahG intensifies the photons emitted from FBP-infiltrated *N. benthamiana* leaves. Autoluminescence images were collected 72 hours after *Agrobacterium* infection. NahG transiently expressed in tobacco leaves was determined by immunoblot analyses. Values are mean ± SD (*n* = 3). Statistical significance was assessed using two-tailed Student's *t*-tests (^**^P ≤ 0.01). (B) Subcellular localization of GFP-tagged NahG in *N. benthamiana* leaves, NLS-mCherry was used as nuclear marker. Scale bars, 20 μm. (C) LC–MS/MS analysis of the content of SA from injected tobacco leaves. Values are mean ± SD (*n* = 3). Statistical significance was assessed using two-tailed Student's *t*-tests (^**^P ≤ 0.01). (D) P19 enhances the expression of FBP in infiltrated *N. benthamiana* leaves, autoluminescence intensity and protein expression analyses were carried out 72 hours after *Agrobacterium* infection. Western blot analysis was conducted using GFP antibody. Values are mean ± SD (*n* = 3). Statistical significance was assessed using two-tailed Student's *t*-tests (^**^P ≤ 0.01). (E) Subcellular localization of P19 fused to GFP in *N. benthamiana* leaf cells, NLS-mCherry was used as nuclear marker. Scale bars, 20 μm. (F) Analysis of agroinfiltration enhancement of P19 at different concentrations, autoluminescence intensity analysis was conducted 72 h after *Agrobacterium* infection. The OD_600_ values of P19 shown above are labeled as the final concentration of the infection solution. Values are mean ± SD (*n* = 3). Statistical significance was assessed using two-tailed Student's *t*-tests (^**^P ≤ 0.01). (G–I) Analysis to determine the enhancement effect of NahG and various concentrations of P19 in agroinfiltration. Autoluminescence imaging (G), photon analysis (H), and RT-qPCR assays (I) on *H3H* and *Luz* were performed 72 hours after infection with *Agrobacterium*. NaP19 indicating the NahG-P2A-P19. (J) NahG expression alleviates phytotoxicity induced by high concentrations of P19 in agroinfiltration. Scale bars, 1 cm. Values are mean ± SD (*n* = 3). Statistical significance was assessed using two-tailed Student's *t*-tests (^*^P ≤ 0.05, ^**^P ≤ 0.01). (K) Leaf yellowing and cell death in tobacco induced by P19 and NaP19 at increasing *Agrobacterium* infiltration concentrations. Symptoms were photographed at 3 days postinfiltration (dpi). (L) Trypan blue staining of necrotic responses in tobacco leaves at 3 dpi. The extent and intensity of blue staining within the infiltrated area correspond to the severity of cell death, Scale bars, 1 cm. (M) Quantification of leaf chlorosis and cell death induced by different combinations of infiltration factors and bacterial concentrations. Lesion ratios were calculated from 20 biological replicates (*n* = 20). Visual damage was scored according to the immune response grading system for *N. benthamiana* established by Xi *et al.* [76]. (N) LC–MS/MS analysis of SA content in infiltrated tobacco leaves. Data are presented as mean ± SD (*n* = 3). Statistical significance was determined by two-tailed Student's *t*-test (^**^P ≤ 0.01).

We employed an FBP-based reporter system in *N. benthamiana* to screen enhancers of *Agrobacterium*-mediated transient transformation. Based on prior evidence that SA suppresses transgene expression [37], we investigated the SA-hydroxylase NahG. Agroinfiltration of an FBP reporter with NahG significantly increased reporter expression compared to the FBP-only control (Fig. 2A). Confocal microscopy confirmed the successful expression and subcellular localization of NahG in the nucleus and cytoplasm of plant cells (Fig. 2B). To establish a direct mechanistic link, we quantified SA levels and found that NahG infiltration resulted in a significant reduction of SA content (Fig. 2C). Collectively, these results demonstrate that NahG enhances transient transgene expression by degrading SA and thereby attenuating SA-dependent plant immune responses.

Based on their known roles in suppressing plant immunity, we assessed whether the *Pseudomonas syringae* Type III effectors AvrPto, AvrPtoB, HopF2 [38], and the chromatin remodeling factor GIF1 could enhance *Agrobacterium*-mediated transient transformation [39]. In contrast to the strong enhancement observed with NahG, none of these effectors significantly increased the transient expression of the FBP reporter in agroinfiltrated *N. benthamiana* leaves (Fig. S3A–D). This lack of enhancement was consistent across different experimental combinations and was further validated in other plant species, including *N. benthamiana* and *Gossypium barbadense* (Fig. S3E and F). Notably, NahG consistently produced a more than 2-fold increase transient transgene expression across tested systems. These results indicate that, unlike in stable transformation, the suppression of specific early defense pathways by these Type III effectors is insufficient to enhance transient T-DNA expression. The unique efficacy of NahG highlights the critical and broad-acting role of SA degradation in facilitating efficient agroinfiltration.

To counteract PTGS during agroinfiltration, we evaluated the co-expression of the tomato bushy stunt virus P19 protein, a known suppressor of RNA silencing that sequesters small interfering RNAs [40]. Co-infiltration of P19 with the FBP reporter in *N. benthamiana* leaves resulted in a significant, 2-fold increase in autoluminescence compared to the FBP-only control (Fig. 2D). Confocal microscopy confirmed the expected nuclear and cytoplasmic localization of P19 (Fig. 2E). Given that high levels of P19 can be detrimental to plant cells [41], to determine the optimal infiltration conditions, we titrated *Agrobacterium* concentrations. An OD600 of 0.25 was found to maximize transgene expression while minimizing phytotoxicity (Fig. 2F). To ensure consistency across all cross-species comparisons, this standardized bacterial density was used in all subsequent agroinfiltration assays. P19 functions through sequence-specific binding to siRNAs, thereby preventing RISC assembly. Although siRNA size classes can vary across plant lineages, such as in monocots and woody plants, P19 is theoretically expected to retain conserved activity throughout the plant kingdom. To experimentally assess the universality of P19 function across diverse plant lineages, we infiltrated P19 into multiple species. Our results demonstrate that P19 exhibits broad-spectrum activity across monocots and woody plants, as evidenced by FBP luminescence intensity and exogenous gene expression levels (Fig. S4A–H). Collectively, these findings indicate that P19, when applied at an optimal concentration, serves as a highly effective tool for enhancing transient expression across multiple plant species.

To simultaneously target SA-dependent defense and RNA silencing, we engineered a dual-effector construct, NaP19, by fusing NahG to P19 via a P2A self-cleaving peptide. Co-expression of the NaP19 construct with the FBP reporter in *N. benthamiana* resulted in a dramatic, 10-fold increase in autoluminescence compared to the FBP-only control, significantly outperforming the expression levels achieved by NahG or P19 alone (Fig. 2G and H). To determine whether the enhanced bioluminescence reflected increased mRNA stability rather than metabolic flux [32, 33], we performed reverse transcription quantitative PCR (RT-qPCR). Transcript levels of the FBP reporter genes *H3H* and *Luz* were significantly upregulated upon NaP19 co-expression (Fig. 2I), indicating that NaP19 increases steady-state mRNA accumulation.

High-level expression of *P19* induces cytotoxicity [41], however, co-expression with *NahG* significantly enhances exogenous gene expression and autoluminescence output (Fig. 2G–I). We hypothesized that *NahG* alleviates *P19*-associated phytotoxicity. To test this, we examined cell death in leaves expressing *P19*, *NahG*, or *NaP19* (Fig. 2J). Cell death was quantitatively assessed using trypan blue staining in *N. benthamiana* leaves infiltrated with *Agrobacterium* harboring *P19*, *NahG*, or *NaP19* across a range of OD600 values (Fig. 2K). At 3 days postinfiltration, leaf discs were stained with trypan blue (Fig. 2L), *P19* alone induced dose-dependent cell death, with significant increases in trypan blue uptake at OD600 ≥ 0.25 compared to empty vector (EV) controls (Fig. 2K–L and Fig. S5A–I). In contrast, *NahG* alone showed no marker difference at any OD600 tested. Notably, co-expression of *P19* with *NahG* (NaP19) significantly reduced cell death across all concentrations (Fig. 2K–L and Fig. S5A–I), confirming that *NahG* mitigates *P19*-associated cytotoxicity in both tobacco and cotton. Consistent with these findings, SA levels were elevated upon high-concentration *P19* expression but were markedly reduced in tissues co-expressing NaP19 (Fig. 2N), suggesting that *NahG* suppresses *P19*-induced SA biosynthesis. Together, these results demonstrate that NahG and P19 synergistically enhance *Agrobacterium*-mediated transient expression by attenuating plant immune responses and stabilizing exogenous mRNA. Furthermore, the NaP19 module mitigates P19-induced cytotoxicity through SA depletion, establishing it as a highly effective enhancer for optimizing transient expression systems in plants.

### NaP19 facilitated agroinfiltration in multiple species

To investigate the effectiveness of NaP19-mediated agroinfiltration in various plant species, we conducted experiments using nine different plant species, including ornamental plants, such as *Adenium obesum*, *Dahlia pinnata*, *Tulipa gesneriana*, *Begonia hiemalis*, *Rhamnus hybrida*, *P. aphrodite*, as well as main crops like *Helianthus annuus*, *G. hirsutum*, and *Vigna radiata*. Despite the importance of these plants in horticulture and agriculture, the use of *Agrobacterium*-mediated transient expression assays has not been well-established, limiting molecular research and genetic improvement efforts in these plants.

**Figure 3:**
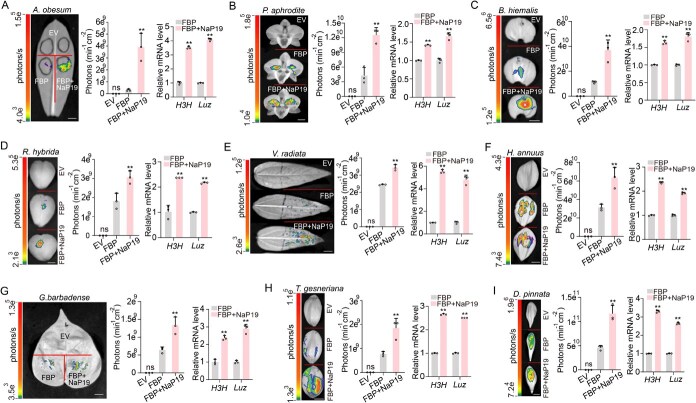
Expression of NaP19 enhances agroinfiltration efficiency across diverse plant species using the FBP-based reporter system. (A–H) Quantification of autoluminescence signal and RT-qPCR analysis of *H3H* and *Luz* transcript levels in tissues infiltrated with EV, FBP alone, or FBP + NaP19. Measurements were taken at 72 hours post-infiltration. Fold changes in photon emission and corresponding *P*-values from plant species include: (A) *A. obesum* leaves (14.21, *P* = 0.00012), (B) *P. aphrodite* petals (3.05, *P* = 0.00537), (C) *B. hiemalis* petals (3.23, *P* = 0.00492), (D) *R. hybrida* petals (1.71, *P* = 0.00894), (E) *V. radiata* leaves (1.39, *P* = 0.00246), (F) *H. annuus* petals (2.06, *P* = 0.00417), (G) *G. hirsutum* leaves (2.14, *P* = 0.0049), (H) *T. gesneriana* petals, (2.36, *P* = 0.00308) and (I) *D. pinnata* petals (2.61, *P* = 0.00191). The tissues were treated with different components, including EV, FBP, and FBP + NaP19 after 72 h. RT-qPCR analysis of *H3H* and *Luz* genes from FBP pathway, reference genes for each species can be found in Table S2. Scale bars, 1 cm. ns, no signal. Values are mean ± SD (*n* = 3). Statistical significance was assessed using two-tailed Student's *t*-tests (^**^P ≤ 0.01).

To evaluate the efficacy of NaP19 in enhancing transient expression, we performed agroinfiltration assays in a diverse set of plant species using *Agrobacterium* strains harboring the FBP reporter, the FBP combined with NaP19, or an EV control. Plants co-infiltrated with FBP and NaP19 exhibited a substantial and significant increase in autoluminescence intensity compared to those infiltrated with FBP alone or the EV control (Fig. 3A–H and Fig. S6A–I), indicating a strong enhancement of reporter activity. This visual enhancement was corroborated at the molecular level by RT-qPCR analysis, which revealed a concomitant significant upregulation of the core FBP genes, *H3H* and *Luz*, confirming that NaP19 boosts transgene expression at the mRNA level (Fig. 3A–I). The strong positive correlation between the accumulation of H3H and Luz transcripts and the intensity of autoluminescence signals further validates the FBP system as a quantitative and reliable reporter for transient expression assays in vascular plants. Collectively, these results demonstrate that the effector protein NaP19 consistently and significantly enhances the efficiency of *Agrobacterium*-mediated transgene expression across a broad phylogenetic range of plant species.

Evaluation of NaP19 efficacy revealed a significant enhancement in transient expression efficiency, supported by increased activity at both the autoluminescence and transcriptional levels (Fig. 3 and Fig. S6A–I). To assess the broad applicability of this system, we extended our analysis across a diverse spectrum of vascular plants. Strong enhancement of autoluminescence was consistently observed in leaves co-infiltrated with *Agrobacterium* carrying both FBP and NaP19, compared to FBP alone, across all tested species (Fig. S7A–I). This included eudicots (*Syzygium grijsii*, *Senecio radicans*, *Ranunculus asiaticus*, *Consolida ajacis*, *Bryophyllum pinnatum*, *Cyclamen persicum*, *Campanula medium*, *Ligustrum ovalifolium*), a monocot (*Dracaena sanderiana*), and was further validated in an expanded panel of species, encompassing gymnosperms (*Nageia nagi*, *Taxus chinensis*) and additional angiosperms including both dicots (*e.g. Rosa odorata*, *Camellia japonica*) and monocots (*Freesia hybrida*) (Fig. S8A–J). The consistent and significant boost in autoluminescence output across this phylogenetically diverse group confirms that the NaP19 system robustly enhances agroinfiltration efficiency throughout vascular plants. These findings underscore the dual utility of the FBP system as a versatile reporter and highlight NaP19 as a potent broad-spectrum effector for advancing transient expression technologies, with significant implications for high-throughput plant research and biotechnology applications.

### Using the agroinfiltration method for *in vivo* assay of *H. annuus* and *V. radiata*

**Figure 4:**
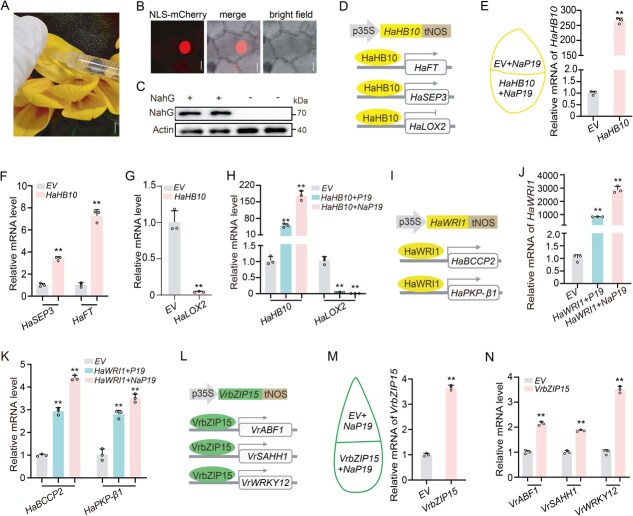
Optimized agroinfiltrated strategies for investigating transcriptional regulation in *H. annuus* and *V. radiata*. (A) Schematic diagram of *H. annuus* petal injection using the syringe. Samples from flat petals of *H. annuus*. Scale bar, 1 cm. (B) Subcellular localization of NLS-mCherry visualized using a Zeiss LSM 880 confocal microscope. Scale bars, 10 μm. (C) NahG from NaP19 transiently expressed in tobacco leaves was determined by immunoblot analyses using GFP antibodies. (D) Schematic diagram of the *HaHB10* expression constructs. HaHB10 acts upstream of *HaSEP3*, *HaFT*, and *HaLOX2*. (E) Relative expression levels of the *H. annuus* transcription factor gene *HaHB10* measured by RT-qPCR in petal tissues agroinfiltrated with an *HaHB10* overexpression construct or an EV control, as illustrated in the diagram. Values are mean ± SD (*n* = 3). Statistical significance was assessed using two-tailed Student's *t*-tests (^**^P ≤ 0.01). (F and G) Relative transcript levels of putative HaHB10 target genes were quantified by RT-qPCR. Expression of upregulated genes (F) *HaSEP3* and *HaFT*, and downregulated gene (G) *HaLOX2* are shown. Values are mean ± SD (*n* = 3). Statistical significance was assessed using two-tailed Student's *t*-tests (^**^P ≤ 0.01). (H) RT-qPCR analysis of relative transcript levels of the sunflower transcription factor gene *HaHB10* and its target gene *HaLOX2* in petal tissues. Petals were agroinfiltrated with an HaHB10 overexpression construct or an EV control, each co-infiltrated with either P19 or NaP19 at a final OD_600_ of 0.25. Data means SD (*n* = 3). Statistical significance was determined by two-tailed Student's *t*-tests (^**^P ≤ 0.01). (I) Schematic representation of the HaWRI1 expression constructs. HaWRI1 functions upstream of *HaBCCP2* and *HaPKP-β1*. (J) Relative transcript levels of *HaWRI1* in petal tissues agroinfiltrated with an *HaHB10* overexpression construct or EV control, each co-infiltrated with either P19 or NaP19 at a final OD_600_ of 0.25. Data means SD (*n* = 3). Statistical significance was determined by two-tailed Student's *t*-tests (^**^P ≤ 0.01). (K) RT-qPCR analysis of *HaBCCP2* and *HaPKP-β1* expression under the same conditions. Data means SD (*n* = 3). Statistical significance was determined by two-tailed Student's *t*-tests (^**^P ≤ 0.01). (L) Schematic model of proposed regulatory interactions between VrbZIP15 and its target genes *VrABF1*, *VrSAHH1*, and *VrWRKY12* in *V. radiata* leaves, as supported by *in vivo* assays. (M and N) RT-qPCR assay of the overexpression of *VrbZIP15* (M) and the upregulation genes *VrABF1*, *VrSAHH1*, and *VrWRKY12* (N) *in vivo*. Values are mean ± SD (*n* = 3). Statistical significance was assessed using two-tailed Student's *t*-tests (^**^P ≤ 0.01).

Building on our successful agroinfiltration of *H. annuus* with the NaP19 enhancer (Fig. 3E and F), we developed a method for *in vivo* functional studies (Fig. 4A). Infiltration with an NLS-mCherry reporter construct confirmed the system's efficacy, yielding robust nuclear fluorescence (Fig. 4B) and verifying successful protein expression and localization in *H. annuus* tissues. Western blot analysis further confirmed the expression of the NahG component of NaP19 (Fig. 4C), establishing a reliable platform for transient assays in this species.

To demonstrate the utility of this system, we investigated the function of the *H. annuus* transcription factor HaHB10, whose study has been hindered by the lack of efficient stable transformation in *H. annuus* [42]. Using NaP19-assisted agroinfiltration (Fig. 4D), we achieved robust transient overexpression of HaHB10 in its native context (Fig. 4E), enabling the rapid identification of candidate direct targets. Transcriptional analysis revealed that HaHB10 significantly upregulated key flowering integrators, *HaSEP3* and *HaFT* (Fig. 4F), demonstrating the utility of this method for rapidly establishing flowering regulatory networks in nonmodel plants. Concurrently, *HaLOX2*, a gene involved in jasmonic acid (JA) biosynthesis, was downregulated (Fig. 4G), functionally validating the previously proposed role of HaHB10 in modulating JA signaling [43]. Given the well-established antagonism between SA and JA signaling [44], we utilized NahG expression to deplete endogenous SA and assess its effect on JA-related gene regulation. Transient co-expression assays revealed that HaHB10 significantly repressed the JA biosynthesis gene *HaLOX2*, and this repression was further enhanced in the presence of NahG (Fig. 4H), supporting a role for HaHB10 in suppressing *HaLOX2* by agroinfiltration assay. To further evaluate the platform's ability to capture native regulatory relationships in a hormonally minimized context, we tested the conserved oil biosynthesis transcription factor HaWRI1. While previous studies in heterologous *Arabidopsis* systems established that HaWRI1 can activate *BCCP2* and *PKP-β1* [45], it remained unclear whether this regulation operates similarly in its native cellular environment. Our transient system resolved this by demonstrating, for the first time in sunflower, that HaWRI1 directly activates its endogenous targets *HaBCCP2* and *HaPKP-β1* (Fig. 4I–K). This confirms the conservation of the regulatory module while providing a functional validation in the native species that was previously lacking.

We further extended the applicability of this approach to another recalcitrant legume crop, *V. radiata*, by investigating an uncharacterized transcription factor, VrbZIP15 (Fig. 4L and M). Overexpression of VrbZIP15 led to the significant upregulation of three putative downstream targets *VrABF1*, *VrSAHH1*, and *VrWRKY12* (Fig. 4N). These factors are linked to stress and development in other species, suggesting that VrbZIP15 may orchestrate a broader transcriptional program. While a direct phenotypic readout, such as stress tolerance, is beyond the immediate scope of this methods-focused study, these data establish a testable hypothesis and provide the foundational molecular evidence for its role, paving the way for future functional studies. Collectively, these results establish NaP19-assisted agroinfiltration as a powerful tool for *in vivo* protein localization and functional gene analysis. By enabling both the rapid dissection of transcriptional networks in their native context and the validation of conserved regulatory modules, this approach provides a fast, high-throughput alternative to stable transformation for functional genomics in nonmodel and recalcitrant plant species.

### NaP19-mediated agroinfiltration *in vivo* assay in multiple cotton cultivated species

**Figure 5:**
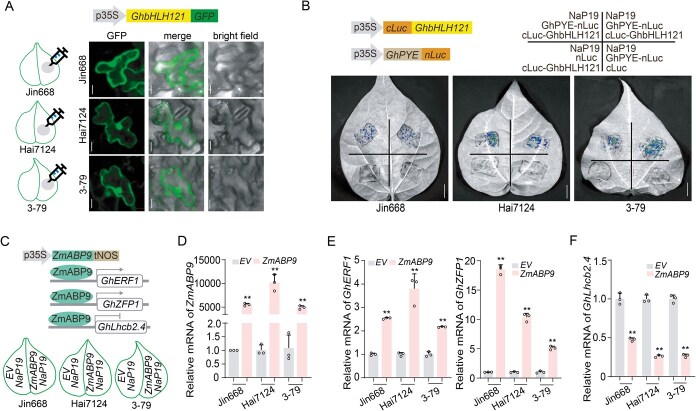
NaP19-mediated transient transformation method for assessing luciferase complementation and transcriptional regulation assay in various cotton species. (A) The subcellular localization of GhbHLH121 in *G. hirsutum*, acc Jin668 and *G. barbadense*, acc Hai7124 and 3-79 were visualized using a confocal microscope. Scale bars, 10 μm. (B) The Luciferase complementation assay of the interactions between GhbHLH121 and GhPYE. Fusion vectors were introduced into cotton leaves for transient coexpression. Scale bar, 1 cm. (C) The schematic illustrations of ZmABP9 stimulate the expression of *GhERF1*, *GhZFP1,* and *GhLhcb2.4*. The injection diagram indicates the agroinfiltration of devious cotton species. EV as control. (D–F) RT-qPCR assay of the expression of *ZmABP9* (D) upregulated genes *GhERF1* and *GhZFP1* (E), downregulated gene *GhLhcb2.4* (F) in agroinfiltrated cotton leaves. Values are mean ± SD (*n* = 3). Statistical significance was assessed using two-tailed Student's *t*-tests (^**^P ≤ 0.01).

To address the challenge of genotype-dependent transformation in cotton (*Gossypium* spp.), we applied our optimized NaP19-mediated agroinfiltration method, which significantly enhanced transformation efficiency across multiple cotton genotypes (Fig. 3G). We successfully expressed a GhbHLH121–GFP fusion protein in three distinct allotetraploid cultivars, *G. hirsutum* (Jin668) and *G. barbadense* (Hai7124, 3–79), observing consistent nuclear and cytoplasmic localization in all cases (Fig. 5A), confirming previous observations obtained with this agroinfiltration method in tobacco leaf assays [46]. Leveraging this efficient system, we investigated a key protein–protein interaction in the iron deficiency response [46]. Using a luciferase complementation assay, we demonstrated a direct interaction between the iron-regulatory transcription factors GhbHLH121 and GhPYE in the native cotton cellular environment (Fig. 5B). These results establish NaP19-assisted agroinfiltration as a robust and genotype-flexible platform for transient functional studies in cotton, enabling critical assays such as protein subcellular localization and *in vivo* protein–protein interaction analysis.

To further validate our transient system for dissecting transcriptional networks, we investigated the function of the maize transcription factor ZmABP9, a known enhancer of abiotic stress tolerance [47]. Using NaP19-mediated agroinfiltration (Fig. 5C), we transiently expressed *ZmABP9* in three distinct cotton genotypes and confirmed its overexpression by RT-qPCR (Fig. 5D). Consistent with findings from stable transgenic cotton overexpressing *ZmABP9* [48], our study revealed significant upregulation of the target genes *GhZFP1* and *GhERF1* across all three accessions (Fig. 5E). Notably, while the direction of regulation was conserved, the magnitude of induction was 2 to 3-fold lower than that reported in stable transgenic lines [48]. This quantitative difference may be attributed to variations in genetic background and to the transient nature of agroinfiltration, where only a subset of mesophyll cells express the transgene, in contrast to the constitutive expression achieved in stable transformants [49]. Beyond validating known targets, our transient system revealed an additional layer of regulation, Lhcb2.4, which encodes a light-harvesting chlorophyll a/b-binding protein [50], was significantly downregulated upon ZmABP9 expression (Fig. 5F). This repression suggests that ZmABP9 may orchestrate a broader physiological shift, balancing growth, and stress responses. These findings position ZmABP9 within a more complex regulatory network that integrates stress signaling with photosynthetic modulation, extending prior knowledge of its function. Collectively, these results demonstrate that the NaP19-mediated transient expression system is a highly efficient and sensitive tool for rapid gene characterization. Its consistent performance across diverse genetic backgrounds establishes it as a versatile platform for functional genomics in a broad range of plant species.

## Discussion

Agroinfiltration is a cornerstone technique for transient gene expression, yet its utility has been largely confined to model systems like *N. benthamiana*. Expanding this method to a broader range of plant species requires both optimized protocols and a universal, quantitative reporter system. Conventional reporters such as GUS, luciferase, and fluorescent proteins are valuable tools but are limited by labor-intensive protocols and the need for invasive quantitative detection methods [51–53]. While the newer RUBY system offers a noninvasive, cost-effective alternative [28, 54], it is constrained by sensitivity and quantification challenges in many plant species [26, 55].

The FBP presents a transformative alternative. The pathway's reliance on caffeic acid [29, 35], a metabolite with evolutionarily conserved biosynthesis across land plants (Figs 1, S1, and  S2), suggests it can harness endogenous substrate pools, functioning without exogenous application. This study successfully demonstrates the FBP's broad applicability, enabling quantitative autoluminescence detection across diverse plant species using a sensitive CCD camera, without the need for substrates or external light excitation. Our results (Figs 3 and S4–S6) establish the FBP as a superior reporter for agroinfiltration. It provided a sensitive and quantitative measure of transformation efficiency, representing an alternative to traditional GUS and GFP systems. This high sensitivity was critical for the accurate screening of enhancing factors, such as NaP19, and effectively minimizing false negatives. By providing a robust and versatile readout, the FBP system overcomes a key bottleneck, paving the way for advanced molecular pharming and functional genomics studies in a wide spectrum of plant species. Despite its autoluminescence advantage, the FBP readout is inherently dependent on the endogenous caffeic acid pool, which can fluctuate under stress or metabolic perturbation. This limitation necessitates rigorous experimental controls and orthogonal validation, such as the RT-qPCR analyses employed in this study. We acknowledge that an internal control independent of phenylpropanoid metabolism would further strengthen quantitative rigor. Future efforts will therefore focus on engineering FBP variants with reduced sensitivity to precursor fluctuations, toward a more robust platform for quantitative imaging.

Agroinfiltration inevitably triggers a basal plant immune response, analogous to pattern-triggered immunity, which significantly constrains transformation efficiency [56, 57]. While bacterial Type III effectors like AvrPto can enhance stable transformation in some species [58], we found that several, including AvrPto, AvrPtoB, and HopF2, did not improve transient agroinfiltration efficiency (Fig. S3). This distinction underscores fundamental differences in the biological constraints between stable genetic modification and transient expression. Our results demonstrate that targeting key, broad-acting defense hubs is a more effective strategy for transient transformation. SA is a central phytohormone that amplifies local immunity and systemic resistance [59, 60]. We showed that expressing the SA hydroxylase NahG significantly reduced SA accumulation and concurrently enhanced transient gene expression (Fig. 2A–C), confirming that SA-mediated signaling is a major barrier to efficient transient gene expression. It is important to note that because FBP autoluminescence output depends on caffeic acid, a key phenylpropanoid intermediate, modulation of the SA pathway could potentially influence caffeic acid flux through metabolic crosstalk [30, 61]. Although our data demonstrate robust and consistent reporter activity under standardized conditions, this potential interaction warrants consideration when interpreting FBP-based results, particularly in studies involving SA pathway manipulation or when other experimental factors may perturb the dynamic FBP metabolic pathway.

Agroinfiltration often triggers a PTGS response, leading to the destruction of transgene-encoded mRNA [62, 63]. We confirmed that co-expression of the silencing suppressor P19 potently enhanced transgene expression (Fig. 2D–F). However, the utility of P19 is concentration-dependent; we determined an optimal OD600 of 0.25 to maximize yield while avoiding the phytotoxicity associated with its prolonged expression (Fig. 2F). Crucially, we found that co-expressing NahG, which alleviates P19 toxicity by suppressing the SA synthesis pathway, acted synergistically with P19 to produce a dramatic, multifold increase in transient expression over either component alone (Figs 2J–N and 3). This synergistic effect demonstrates that plant defenses operate through multiple, parallel pathways. Therefore, an integrated strategy that concurrently mitigates both phytohormone-triggered immunity and RNA silencing is essential for achieving maximal transient expression, providing a robust and widely applicable platform for advanced plant biotechnology.

Transient expression systems like protoplast transformation and agroinfiltration are indispensable for rapid gene functional analysis. However, protoplast isolation is technically demanding and can compromise cellular integrity, limiting its application for *in vivo* studies [1, 64, 65]. While agroinfiltration is simpler, its efficiency has been largely restricted to model species like *N. benthamiana [**9**]*, presenting a major bottleneck for functional genomics in crops. To overcome this limitation, we developed a robust transient expression platform combining a highly sensitive FBP reporter with the potent enhancer module NaP19. This system dramatically improved transformation efficiency across a diverse panel of plant species, including previously recalcitrant horticultural plants and perennial trees (Figs 3 and S4–S6). We demonstrated its practical utility by successfully applying it to analyze transcriptional regulation and protein–protein interactions in key cash crops, such as *H. annuus*, *V. radiata*, and multiple cotton varieties, effectively bypassing genotype-dependent limitations (Figs 4 and 5). Beyond confirming transcriptional activation in native backgrounds, these proof-of-concept assays establish a foundation for linking gene expression changes to downstream phenotypic or metabolic outcomes. Such investigations would enable high-throughput gene function discovery and metabolic pathway mining, highlighting the platform's potential for plant synthetic biology. Its versatility extends to the construction and testing of complex genetic circuits, and integration with CRISPR-based activation or interference technologies offers a powerful strategy for reprogramming metabolic pathways to produce high-value, plant-specific compounds in heterologous systems [66, 67].

A consideration for quantitative work is that the FBP signal, while highly sensitive, is ultimately dependent on cellular metabolism [68]. We note that rigorous experimental controls are essential to account for potential variations in the endogenous metabolic microenvironment when interpreting luminescence data [26]. Despite this, the FBP represents a significant advancement in tracing technology. It offers superior sensitivity and robust quantification while operating without costly substrates or external excitation light. This unique combination facilitates real-time and noninvasive monitoring, thereby overcoming critical limitations of traditional approaches. To enable direct cross-species comparisons, this study employed a standardized agroinfiltration protocol across all species. While this approach ensured experimental consistency, it precluded species-specific optimization of parameters such as *Agrobacterium* density, leaf age, tissue *accessibility*, and infiltration buffer composition. We acknowledge this as a limitation and suggest that tailoring these conditions to individual species may further enhance transient expression efficiency.

In conclusion, our optimized agroinfiltration system provides a rapid, efficient, and species-flexible alternative to stable transformation and protoplast assays. By integrating the quantitative autoluminescence reporter FBP with a synergistic enhancer NaP19, this toolkit offers a powerful platform to accelerate gene characterization and expand both basic research and applications across diverse plant species, particularly those that are nonmodel or recalcitrant.

## Materials and methods

### Plasmid construction

In this study, we generated the plasmids used by sequentially cloning various genetic elements into the pCAMBIA1300 vector. First, the 35S promoter (p35S) and NOS terminator (tNOS) sequences were inserted into the pCAMBIA1300 plasmid using Hind III/Xba I and Sac I/EcoR I restriction enzymes, respectively. Next, the *GFP* fragments were added to the pCAMBIA1300–p35S vector using BamH I/Sac I enzymes, resulting in the generation of pCAMBIA1300–p35S–GFP plasmid. The *P19* gene (GenBank ID: AJ288942.1) from tomato bushy stunt virus and the *NahG* gene (GenBank ID: M60055) from *Pseudomonas putida* were then amplified and connected using overlap extension PCR with a 2A peptide linker. Subsequently, the amplified *P19* and *NahG* fragments were seamlessly cloned into the pCAMBIA1300-p35S vector using 2X MultiF Seamless reagent (ABclonal, USA), resulting in the formation of pCAMBIA1300–p35S–NahG–GFP, pCAMBIA1300–p35S–NahG, pCAMBIA1300–p35S–P19, pCAMBIA1300–p35S–P19–GFP, and pCAMBIA1300–p35S–NahG–P2A–P19 plasmids. Similarly, the pCAMBIA1300–p35S–HaHB10, pCAMBIA1300–p35S–HaWRI1, pCAMBIA1300–p35S–ZmABP9, pCAMBIA1300–p35S–GhbHLH121, and pCAMBIA1300–p35S–VrbZIP15 constructs were generated using the same cloning methodology. To construct vectors that enhance infection, fragments, such as p35S–AvrPto–tNOS and p35S–AvrPtoB–tNOS were inserted into the pCAMBIA1300–NahG–GFP using the HindIII restriction enzyme. Similar procedures were followed for the pCAMBIA1300–HopF2–NahG, pCAMBIA1300–AvrPphe–NahG, and pCAMBIA1300–GIF1–NahG vectors. In the case of the three factor vectors, the HopF2 fragment was inserted into the pCAMBIA1300–AvrPtoB–NahG using the KpnI restriction enzyme.

The FBP construct used in this study is identical to that described in our previous work [31]. It was assembled via the TransGene Stacking II system [69] and comprises the following codon-optimized genes: *Luz*, *HispS*, *H3H*, and *CPH* from *Neonothopanus nambi*, along with *NPGA* from *Aspergillus nidulans*. The primer sequences utilized in the plasmid constructions can be found in Table S2, the vectors' information is shown in Table S3.

### *Agrobacterium*-mediated *Physcomitrium patens* transformation

In the simplified transformation process for *P. patens*, 5 ml of *Agrobacterium tumefaciens* strain EHA105 culture was directly added to each culture dish for a 30-minute treatment, the bacterial solution was removed using a sterile pipette and the culture plate was sealed with Parafilm. The gametophytes were then transferred to a 50-ml centrifuge tube. Subsequently, 20 ml of infection solution was added to the tube, after 30 minutes the mixture was returned to the culture plate. The culture plates were sealed and placed in the dark for 3 days. Following this, the cultures were transferred to standard *P. patens* culture conditions for further growth. Validation was conducted one month after screening and culture to confirm the successful transformation of FBP in *P. patens*.

### Plant material resources preparation

A total of 42 species were included in this study, categorized as 1 algae (*C. reinhardtii*), 2 bryophytes (*P. patens, M. polymorpha*), 1 lycophytes (*D. complanatum*), 1 ferns (*C. richardii*), 3 gymnosperms (*G. biloba*, *N. nagi*, and *T. chinensis*), 2 ANA basal angiosperms (*A. trichopoda, N. colorata*), 32 angiosperms (*N. tabacum*, *A. thaliana*, *O. sativa*, *E. aureum*, *T. erecta*, *R. odorata*, *S. lycopersicum*, *H. annuus*, *Rosa hybrida*, *C. japonica*, *M. figo*, *F. hybrida*, *G. hirsutum*, *G. barbadense*, *P. serrulata*, *V. radiata*, *A. obesum*, *T. gesneriana*, *B. hiemalis*, *P. aphrodite*, *D. pinnata*, *S. grijsii*, *D. sanderiana*, *R. asiaticus*, *C. persicum*, *C. medium*, *S. radicans*, *B. pinnatum*, *C. ajacis*, *N. benthamiana*, *C. roseus*, *L. ovalifolium*). For the plants shown in Fig. 3, specific germplasm resource are: *A. obesum* (Huanshanv), *T. gesneriana* (Virgin), *B. hiemalis* (Huangse Ligehaitang), *R. hybrid* (Juicy Terrazza), *P. orchids* (Baitiane), *H. annuus* (Vincent 2), *G. barbadense* (Hai7124), *V. radiata* (Liaolv 27), *D. pinnata* (Huanxiang). For the plants shown in the attached figure, they are all locally cultivated varieties from southern China.

### Database search

To identify candidate genes involved in the caffeic acid synthesis pathway, multiple database searches were conducted. Initially, the amino acid sequences of key genes (*4CL*, *C3*′*H*, *C3H*, *C4H*, *CSE*, *HCT*, *PAL*) were retrieved from the TAIR database [70]. These sequences were then used as query sequences in BLASTP searches (E value <1e^−2^) to identify additional candidate proteins in Table S1. The candidate genes were obtained from NCBI (https://www.ncbi.nlm.nih.gov/), Ginkgo DB (https://ginkgo.zju.edu.cn/) and Phytozome V13 (https://phytozome-next.jgi.doe.gov/) [71]. Genes that did not show significant similarity to known genes involved in caffeic acid synthesis were removed from the candidate list based on the blast results and domain analysis from online database. This rigorous approach ensured that only relevant candidate genes were considered for further analysis in our study.

### Construction of the phylogenetic tree

The protein sequences of the seven genes involved in the caffeic acid synthesis pathway were aligned using MAFFT with default parameters [72]. Subsequently, phylogenetic trees for each gene were constructed separately using FastTree software, with 1000 resamples and the JTT model for maximum-likelihood analysis [73]. The accuracy of the phylogenetic trees was verified using the MEGA11 tools [74]. All amino acid sequences used for phylogenetic and alignment analysis in this resource study are provided in Table S1.

### *Agrobacterium*-mediated transient transformation in plants

Plasmids were transferred into *A. tumefaciens* strain EHA105, which was cultured in LA Solid medium with 50 mg/l rifampicin and 50 mg/l kanamycin sulfate at 28°C for 48 h. The bacteria were then scraped and resuspended in a 500-ml washing solution containing 10 mM MgCl_2_ and 100 μM acetosyringone to reach an OD_600_ of 0.8 to 1.0. After centrifugation, the bacteria were resuspended in a liquid infiltration medium (1/4 MS salts, 1% sucrose, 100 μM acetosyringone, 0.005% Silwet L-77, pH 5.8) to the OD_600_ range of 0.8 to 1.0 for agroinfiltration. For optimal agroinfiltration, NaP19 (NahG-P2A-P19) and FBP strains were mixed to final OD600 values of 0.25 and 0.8, respectively, into various plant tissues (leaves or petals in the figure legend) in this study. This ratio maximizes expression yield while minimizing phytotoxicity associated with prolonged NaP19 expression. The bacterial suspensions were incubated at room temperature without shaking for 2 hours before use. Using a 1-ml plastic syringe, *Agrobacterium* solution was infiltrated into plant tissue after making a shallow puncture. For infiltration, we selected leaves or flowers that are readily accessible for syringe-mediated *Agrobacterium* delivery. The tissues were allowed to dry in the light for 1 hour, followed by incubation in the dark at room temperature for 12 hours. Transformed plants were then moved to the greenhouse and grown for an additional 2 to 3 days before sample observation. All cotton species were used for agroinfiltration at 20 days after seed germination, with the first true leaf. Mung bean plants, on the other hand, were injected on the first true leaves at 10 days after seed germination.

### Western blot analysis

Tobacco leaves transiently expressing fusion proteins were first homogenized in liquid nitrogen using a mortar and pestle. Each 0.1 g sample of homogenized leaves was then mixed with 200 μl of extraction buffer containing 50 mM Tris–HCl (pH 7.5), 150 mM NaCl, 0.5% TritonX-100, and Roche cocktail protease inhibitor. The mixture was centrifuged at 12 000 g at 4°C for 10 minutes, after which the resulting supernatant was carefully transferred to a new tube. To this supernatant, the one-third volume of 4× Laemmli buffer was added, which consisted of 250 mM Tris–HCl (pH 6.8), 8% SDS, 40% glycerol, 4% β-mercaptoethanol, and 0.01% bromophenol blue. The mixture was then thoroughly mixed and denatured at 98°C for 10 minutes to obtain the denatured mixture containing the total proteins. Subsequently, the total proteins were separated by sodium dodecyl sulphate-polyacrylamide gel electrophoresis (SDS-PAGE) and transferred onto a polyvinylidene difluoride membrane (Millipore, USA). The membrane was then incubated with antibodies against GFP tags, while actin antibody was used as a loading control. The proteins were detected using ECL Western Blotting Detection Reagents (Sparkjade, China).

### LC–MS/MS analysis

The plant samples were harvested and immediately frozen in liquid nitrogen. They were then subjected to lyophilization in 50-ml Falcon tubes. Approximately 100 mg dry weight of lyophilized powder was transferred to 5 ml of extraction buffer containing 70% methanol. The extracts were then subjected to ultrasonication in a water bath for 30 minutes, followed by centrifugation at 13 000 g for 15 minutes. The supernatants were filtered through a PVDF syringe filter with a pore size of 0.45 μm. The filtered extracts were transferred to glass vials for LC/MS analysis, as described in more detail previously [31].

For SA quantification, leaves were harvested three days post-agroinfiltration. Approximately 50 mg dry weight of lyophilized powder was transferred to extraction buffer containing 1 ml ethyl acetate and 100 ng D4-SA as an internal standard. The mixture was vortexed for 10 minutes, then centrifuged at 13 000 rpm for 20 minutes at 4°C. The supernatant was collected, vacuum-dried at 30°C, and the residue resuspended in 500 μl of MeOH:H₂O (70:30, v/v). Following centrifugation at 13 000 rpm for 10 minutes, samples were analyzed by high-performance liquid chromatography-tandem mass spectrometry (HPLC-MS/MS) as previously described [75]. Calibration curves were generated using authentic SA standards over a concentration range of 0.05 to 100 ng/ml (*R*^2^ = 0.998). Each treatment was replicated three times.

### Reverse transcription quantitative PCR

To extract RNA, all leaves were initially flash frozen in liquid nitrogen and then homogenized using VeZol reagent (Vazyme Biotech, China). Subsequently, the 1-μg extracted RNA was utilized to synthesize first-stranded cDNA with the MonScriptTM RTIII Super Mix with dsDNase (Monad Biotech, China), following the manufacturer's protocol. For RT-qPCR analysis, gene transcript levels were determined using SYBR Premix and gene-specific primers on a LightCycler480 II Real-Time PCR machine (Roche, Switzerland). The PCR program included an initial step at 95°C for 1 minutes, followed by 40 cycles of 95°C for 10 seconds, 60°C for 20 seconds, and 72°C for 20 seconds. To ensure reliability, a minimum of three biological replicates were examined for each sample. Relative gene expression was calculated using the 2^ΔΔCt^ method. Primer amplification efficiencies were validated by standard curve analysis prior to experimental runs. The reference genes *NtEF1a, VrActin*, *GhUBQ7*, *HaEF1a*, *Ao5Srrna*, *TgUBQ10*, *PaUbi*, *BhACT2*, *DsActin*, and *RhUbi* were employed for normalizing gene expression, and the primers utilized for amplification are detailed in Table S2.

### Subcellular localization

After the pCAMBIA1300–p35S–GhbHLH121–GFP, pCAMBIA1300–p35S–NLS–mCherry, and pCAMBIA1300–p35S–NahG–P2A–P19 were transformed into the *A. tumefaciens* strain EHA105, the resulting vectors were co-transfected into target tissues. After culturing for 72 hours at 28°C, fluorescence signals were observed and captured on a confocal microscope (Zeiss LSM880, Oberkochen, Germany).

### Luciferase complementation assay

*Agrobacterium tumefaciens* strain EHA105 containing the cLuc-GhbHLH121, GhPYE-nLuc, and NaP19 plasmids were co-injected into 20-day-old cotton leaves with the first true leaves. After 2 days, 0.15 mg/ml D-Luciferin solution was injected into the same site using a 1-ml plastic syringe. After a 20-minute dark treatment, the leaves were imaged using a fully automated luminescence imaging system (Tanon5200, Shanghai, China).

### Trypan blue staining

Leaf samples were collected 3d post-infiltration following transient *Agrobacterium*-mediated transformation. Harvested leaves were immersed in a preheated trypan blue staining solution composed of 1 mg/ml trypan blue (Biosharp, China) and a 1:1:1:1 (v/v) mixture of lactic acid (Hushi, China), glycerol (Hushi, China), water-saturated phenol (Sangon Biotech, China), and distilled water. Staining duration was 70 seconds for tobacco leaves and 80 seconds for cotton leaves. Following staining, leaves were briefly rinsed with absolute ethanol to remove excess dye. Destaining was then performed in anhydrous ethanol on an orbital shaker at room temperature for 3 to 5 days, until complete chlorophyll depigmentation was achieved. Images of the stained leaves were captured using a digital camera (Sony Alpha 1, Japan). Leaf damage was visually assessed using a scoring system adapted from Xi et al [76] for evaluating immune responses in *N. benthamiana*. Responses were categorized into five grades: 0 = no response; 1 = weak chlorosis; 2 = strong chlorosis; 3 = patchy cell death; and 4 = strong confluent cell death.

### Bioluminescence imaging and quantification

Bioluminescence imaging was performed using a NIGHTSHADE LB985 system (Germany) equipped with a back-thinned, ultra-sensitive CCD camera. Samples were placed inside a light-tight dark box approximately 40 cm below the camera. Bioluminescence images were captured with a 60-second exposure time, and photon emission was quantified from defined regions of interest corresponding to leaf or petal luminescence areas. The instrument output was initially recorded as photons per second per square millimeter. For consistency with previous publications, values were converted to photons per minute per square centimeter by multiplying raw data by 60 × 100. Following bioluminescence capture, ambient light images were acquired under identical instrument settings. All other parameters were maintained at default values throughout the experiments. Data were exported for subsequent statistical analysis.

### Statistical analyses

The quantitative data presented in this paper is expressed as mean ± SD, obtained from a minimum of three biological replicates. GraphPad Prism 9 software was utilized for data visualization and plotting, while Excel was employed for statistical analysis. Statistical significance was determined using an unpaired two-tailed Student's *t*-test for comparisons between two groups. The specific *P* values are indicated in the figures for reference.

## Supplementary Material

Web_Material_uhag126

## Data Availability

The data supporting the findings of this study are available within the paper figures and the Supplementary Information.
