# Supplementary material for: Expanding agroinfiltration host range with broad-spectrum effectors monitored by autoluminescent reporter
Source: Hortic Res. 2026 Apr 6;13(8):uhag126. doi: 10.1093/hr/uhag126 (PMC13411270; doi:10.1093/hr/uhag126)
Supplement: Web_Material_uhag126 [file web_material_uhag126.zip › supplemental data (clean version).docx]

**Supplemental information**

**
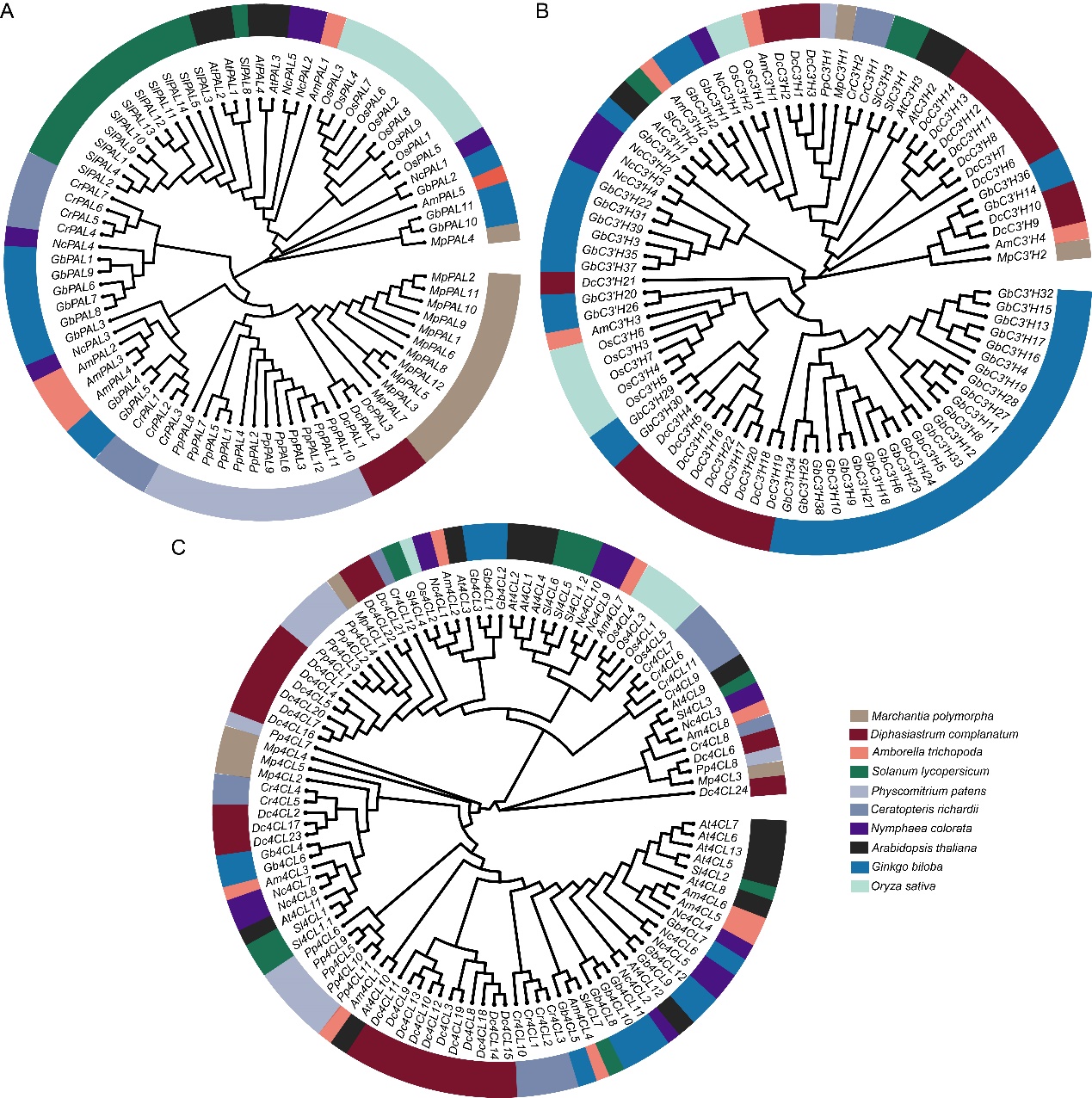
**

**Figure S1. Phylogenetic tree of PAL, C3’H and 4CL in plants orthologs.** The full-length amino acid sequence of PALs (A), C3’Hs (B) and 4CLs (C) from 10 plants species was retrieved with BLASTP (https://phytozome-next.jgi.doe.gov/, https://ginkgo.zju.edu.cn/), including *Marchantia polymorpha* (Map), *Physcomitrium patens* (Php), *Ginkgo biloba* (Gib), *Diphasiastrum complanatum* (Dic), *Ceratopteris richardii* (Cer), *Oryza sativa* (Ors), *Amborella trichopoda* (Amt), *Nymphaea colorata* (Nyc), *Solanum lycopersicum* (Sol) and *Arabidopsis thaliana* (Art). Sequence alignment was used to construct an NJ tree with MEGA 11 (Tamura et al., 2021), using Poisson correction, pairwise deletion, and 1000 bootstrap replicates.


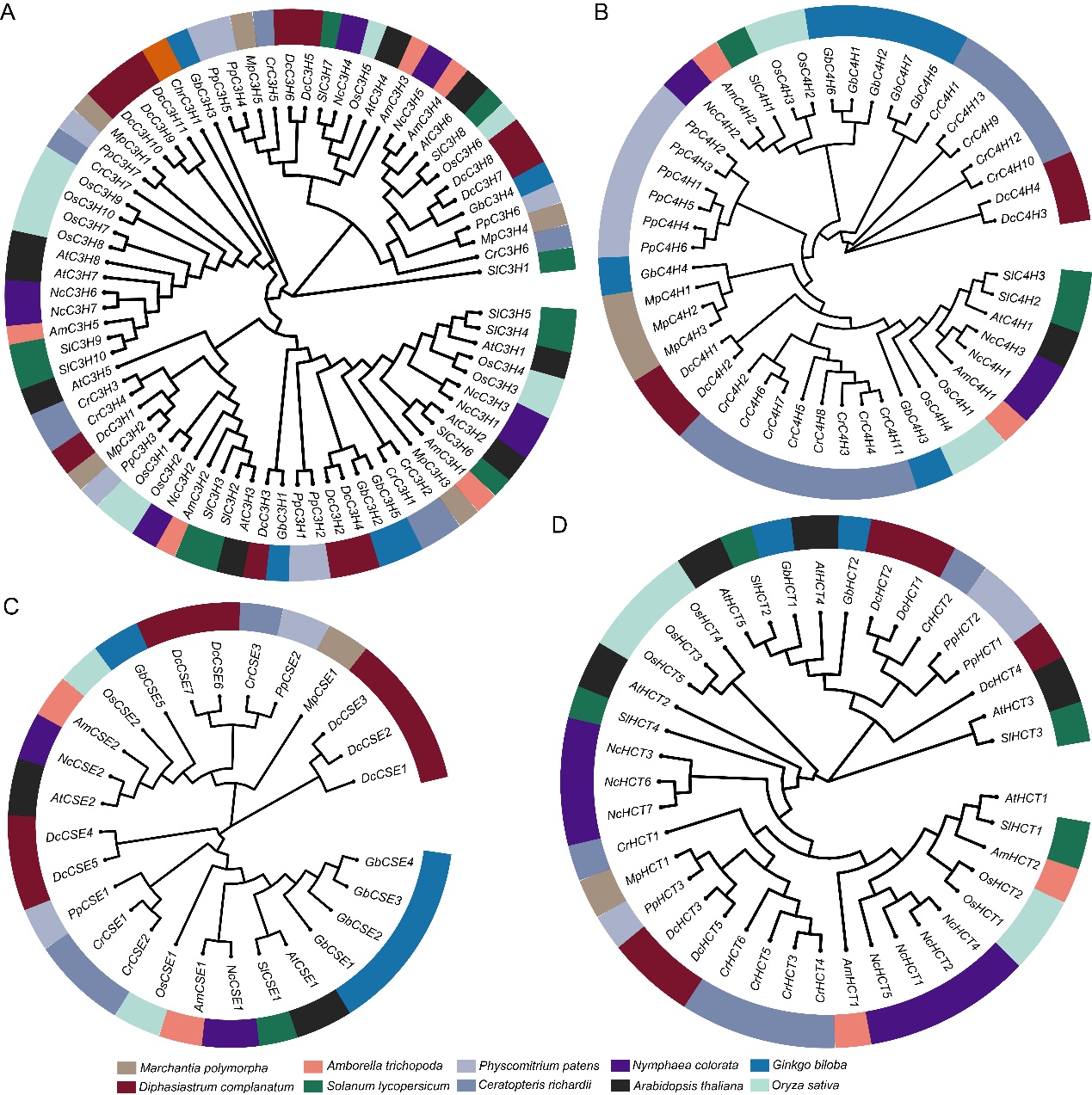


**Figure S2. Phylogenetic tree of C3H, C4H, CSE and HCT in plants orthologs.** The full-length amino acid sequence of C3Hs (A), C4Hs (B), CSEs (C) and HCTs (D) from 10 plants species was retrieved with BLASTP (https://phytozome-next.jgi.doe.gov/, https://ginkgo.zju.edu.cn/), including *Chlamydomonas reinhardtii* (Chr), *Marchantia polymorpha* (Map), *Physcomitrium patens* (Php), *Ginkgo biloba* (Gib), *Diphasiastrum complanatum* (Dic), *Ceratopteris richardii* (Cer), *Oryza sativa* (Ors), *Amborella trichopoda* (Amt), *Nymphaea colorata* (Nyc), *Solanum lycopersicum* (Sol) and *Arabidopsis thaliana* (Art). Sequence alignment was used to construct an NJ tree with MEGA 11(Tamura et al., 2021), using Poisson correction, pairwise deletion, and 1000 bootstrap replicates.


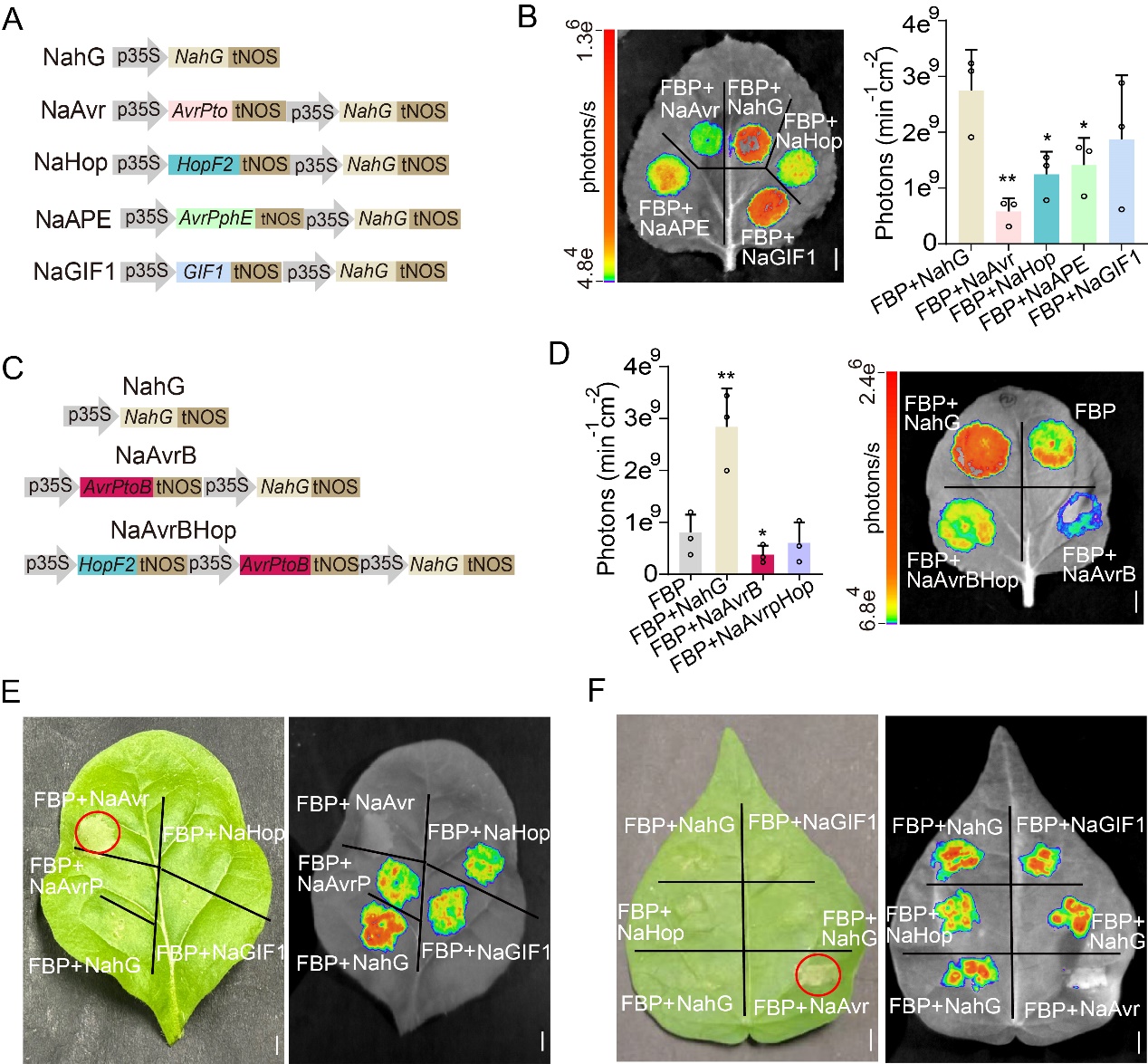


**Figure S3. The screen of enhanced effectors for agroinfiltration efficiency with the FBP-based reporter system in multiple plants.** (A and B) Schematic illustrations (A) and agroinfiltration in *Nicotiana benthamiana* (B) with FBP-based reporter system. NahG (M60055), AvrPto (WP_011104823.1), HopF2 (AAK49537.1), AvrPphE (AAP23130.1), GIF1 (XP_037429183.1). Scale bars, 1 cm. Error bars indicate means ± SD (n = 3). Statistical significance was assessed using two-tailed Student’s *t*-tests (**P* ≤ 0.05, ***P* ≤ 0.01). (C and D) Schematic of agroinfiltration effectors (C) and agroinfiltration in *Nicotiana benthamiana* (D) with FBP-based reporter. Scale bars, 1 cm. Error bars indicate means ± SD (n = 3). AvrptoB (WP_011104378.1). Statistical significance was assessed using two-tailed Student’s *t*-tests (**P* ≤ 0.05, ***P* ≤ 0.01). (E and F) The screen of enhanced effector for agroinfiltration efficiency with FBP-based reporter in *Nicotiana tabacum* (E) and *Gossypium barbadense* (F). red circles indicate the necrotic spots. Scale bars, 1 cm.


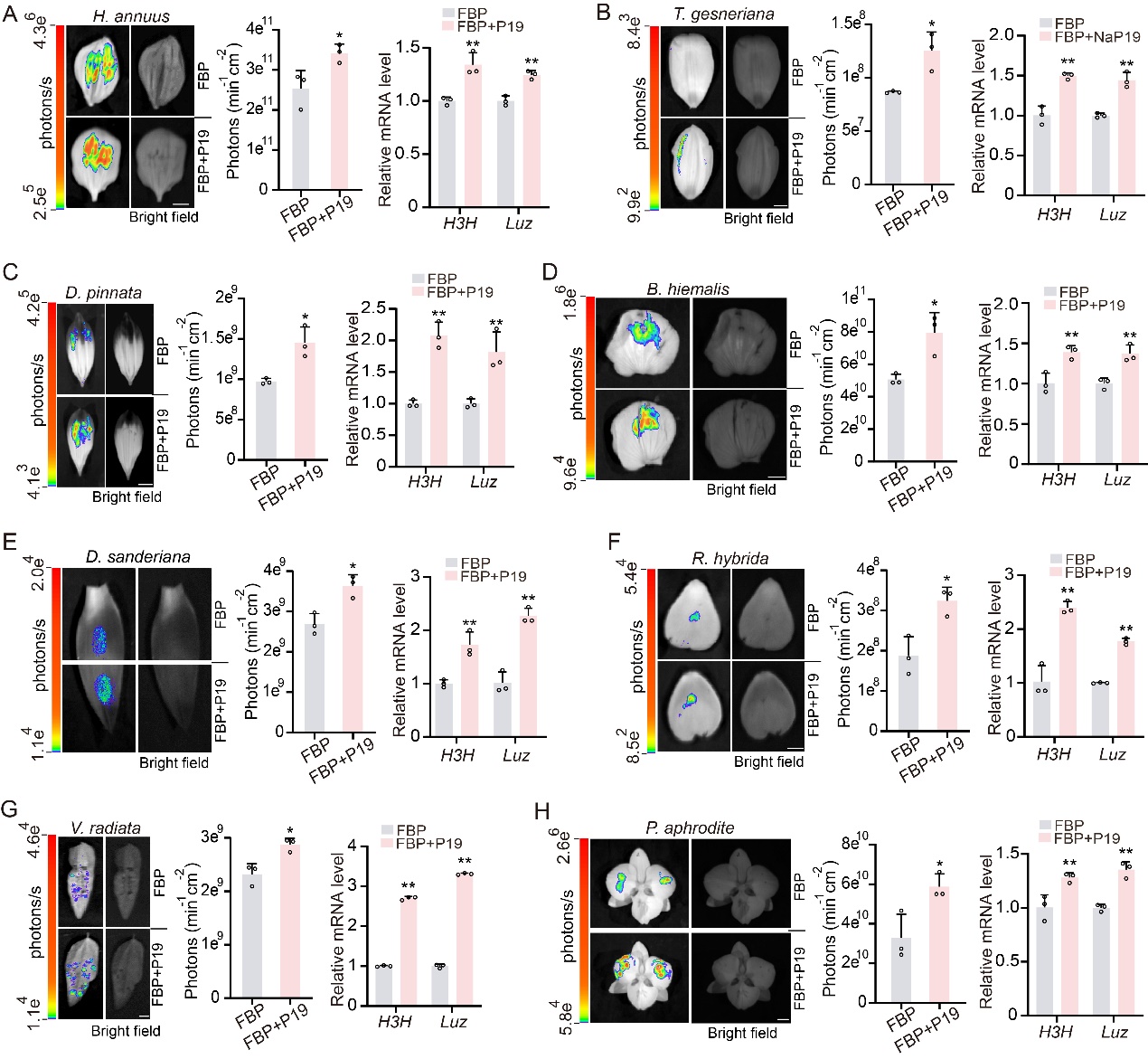


**Figure S4. P19 enhances agroinfiltration efficiency across diverse plant species with the FBP reporter system.** (A-H) Quantification of bioluminescence intensity (left) and RT-qPCR analysis of H3H and Luz transcript levels (right) in leaves of eight plant species agro-infiltrated with FBP alone or FBP + P19. Leaves were harvested 72 hours post-infiltration. Plant species: *Helianthus annuus* (A), *Tulipa gesneriana* (B), *Dahlia pinnata* (C), *Begonia hiemalis* (D), *Dracaena sanderiana* (E), *Rhamnus hybrida* (F), *Vigna radiata* (G), and *Phalaenopsis aphrodite* (H). Reference genes for RT-qPCR normalization are listed in Table S2. Scale bars, 1 cm. Values represent mean ± SD (n = 3). Statistical significance was determined by Student’s t-test (photon counts) and two-way ANOVA with Tukey’s post-hoc test (gene expression); **P* ≤ 0.05, ***P* ≤ 0.01. Fold changes in photon emission and corresponding *P*-values are: *H. annuus* (1.35, *P* = 0.0385), *T. gesneriana* (1.45, *P* = 0.0191), *D. pinnata* (1.49, *P* = 0.0139), *B. hiemalis* (1.57, *P* = 0.0187), *D. sanderiana* (1.35, *P* = 0.0124), *R. hybrida* (1.73, *P* = 0.0155), *V.* *radiata* (1.24, *P* = 0.0114), *P. aphrodite* (1.81, *P* = 0.0451).

**
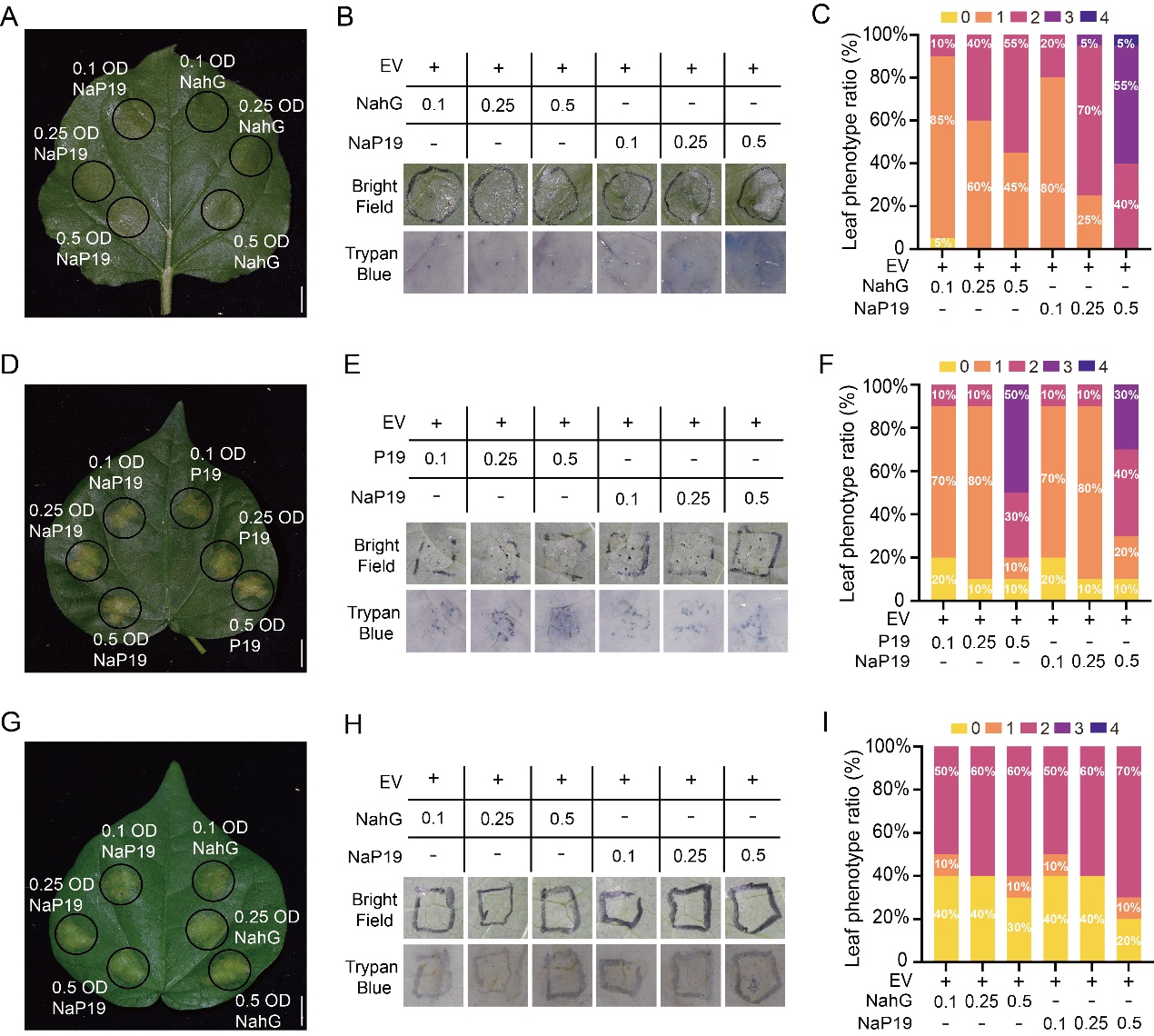
**

**Figure S5. Comparative analysis of P19-induced cell death and alleviated by NahG in tobacco and cotton.** (A-I) Leaves of Nicotiana benthamiana (A-C) and cotton (D-I) were infiltrated with Agrobacterium cultures at indicated OD600 values, alone or in combination with NahG, P19, or NaP19 (P19 + NahG). (A, D, G) Representative images of leaf chlorosis and cell death at 72 hours post-infiltration (hpi). (B, E, H) Trypan blue staining of infiltrated leaf discs at 72 hpi; blue-stained areas indicate necrotic cell death. (C, F, I) Quantification of lesion frequency. Lesion development was scored based on visible chlorosis/necrosis (n = 20 biological replicates for tobacco; n = 10 for cotton). Scoring criteria followed the immune response grading system established by (Xi et al., 2021). Scale bars, 1 cm.


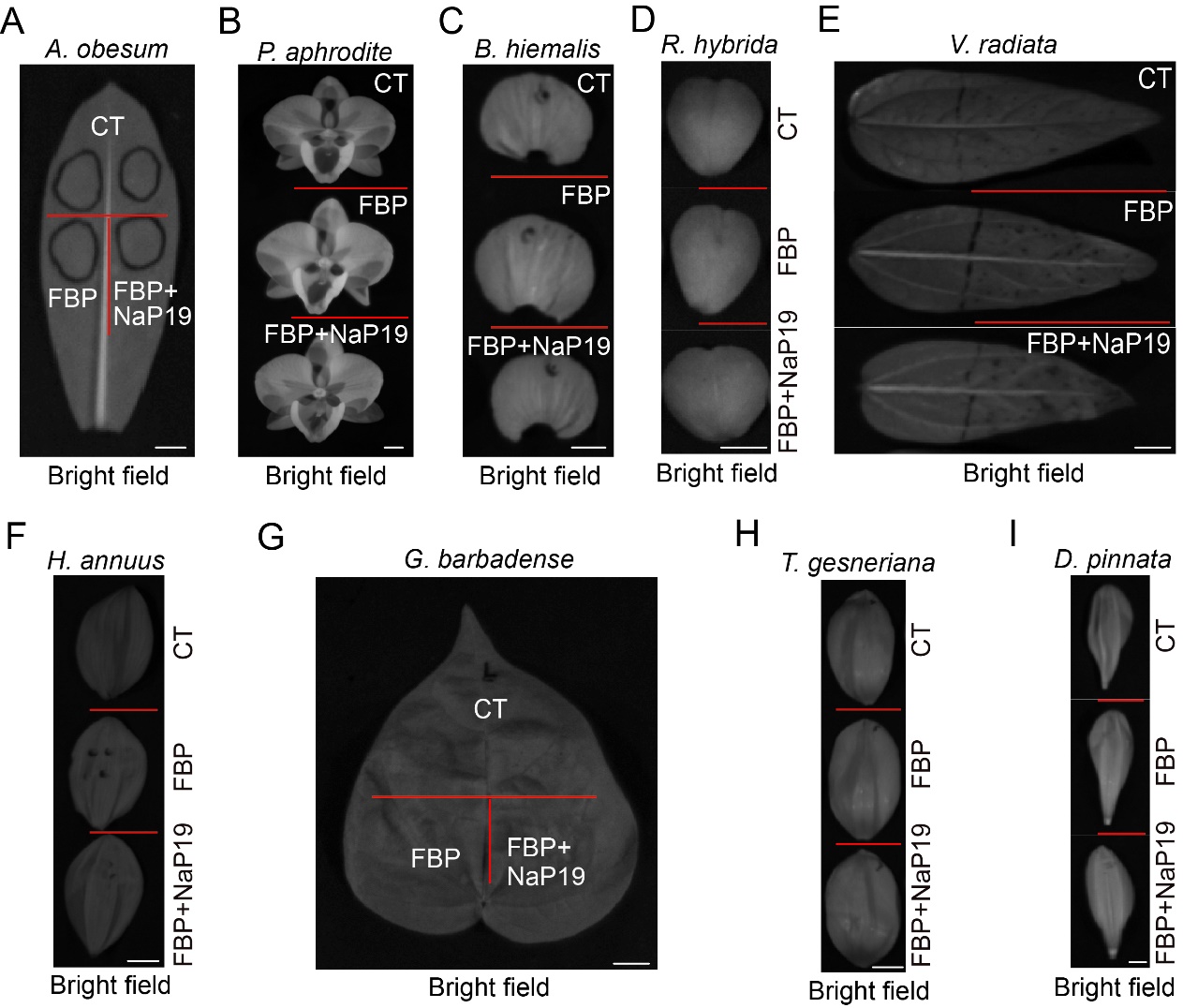


**Figure S6.  Bright-field images of FBP reporter system efficiency across diverse plant species (related to Figure 3).** The bright field image of agroinfiltrated tissues in Fig. 3A-I, *Adenium obesum* (A), *Phalaenopsis aphrodite* (B), *Begonia hiemalis* (C), *Rhamnus hybrida* (D), *Vigna radiata* (E), *Helianthus annuus* (F), *Gossypium barbadense* (G), *Tulipa gesneriana* (H), *Dahlia pinnata* (I), tissues agroinfiltrated with FBP and FBP+NaP19 modules respectively. Bioluminescent intensity analysis from agroinfiltrated leaves after 72 h. Scale bars, 1 cm.

**
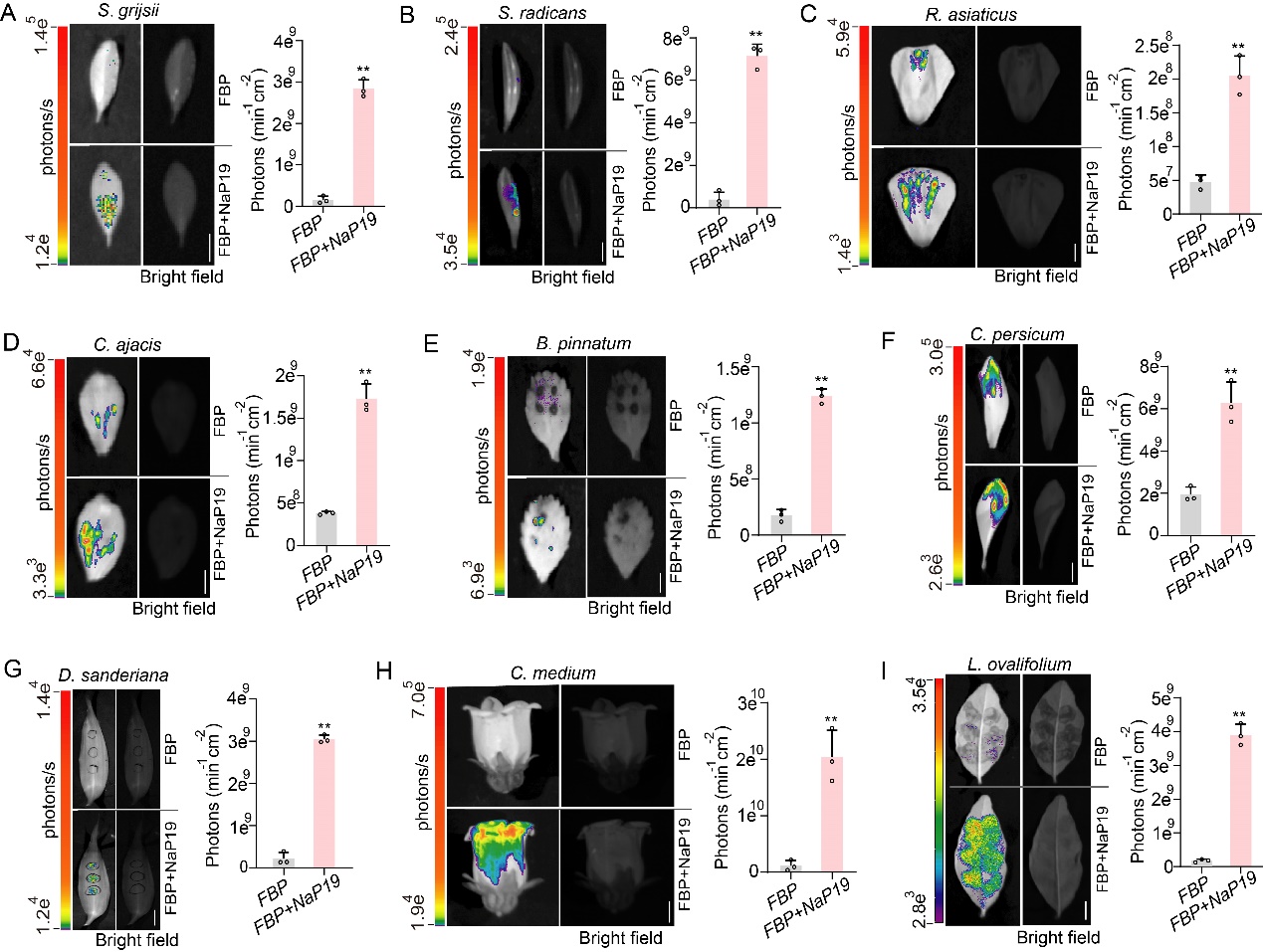
**

**Figure S7. Broad applicability of NaP19 in enhancing agroinfiltration efficiency using the FBP reporter system across diverse plant species.** **(A-I)**Bioluminescence imaging of leaves from nine plant species agroinfiltrated with either the FBP construct alone or FBP + NaP19, captured at 72 hours post-infiltration. Autoluminescence signals were evaluated in various species and tissues at 72 hours post-infiltration with the indicated constructs. Fold changes in photon emission and corresponding *P*-values from plant species include: (A) *Syzygium grijsii* leaves (17.11, *P* = 0.00215), (B) *Senecio radicans* petals (20.37, *P* = 0.00174), (C) *Ranunculus asiaticus* petals (4.18, *P* = 0.00364), (D) *Consolida ajacis* petals (4.51, *P* = 0.0032), (E) *Bryophyllum pinnatum* leaves (6.89, *P* = 0.00277), (F) *Cyclamen persicum* leaves (3.25, *P* = 0.00483), (G) *Dracaena sanderiana* leaves (14.81, *P* = 0.00208), (H) *Campanula medium* petals (20.3, *P* = 0.0327), and (I) *Ligustrum ovalifolium* leaves (22.35, *P* = 0.00153). Scale bars, 1 cm. Error bars indicate means ± SD (n = 3). Statistical significance was assessed using two-tailed Student’s *t*-tests (**P* ≤ 0.05, ***P* ≤ 0.01).


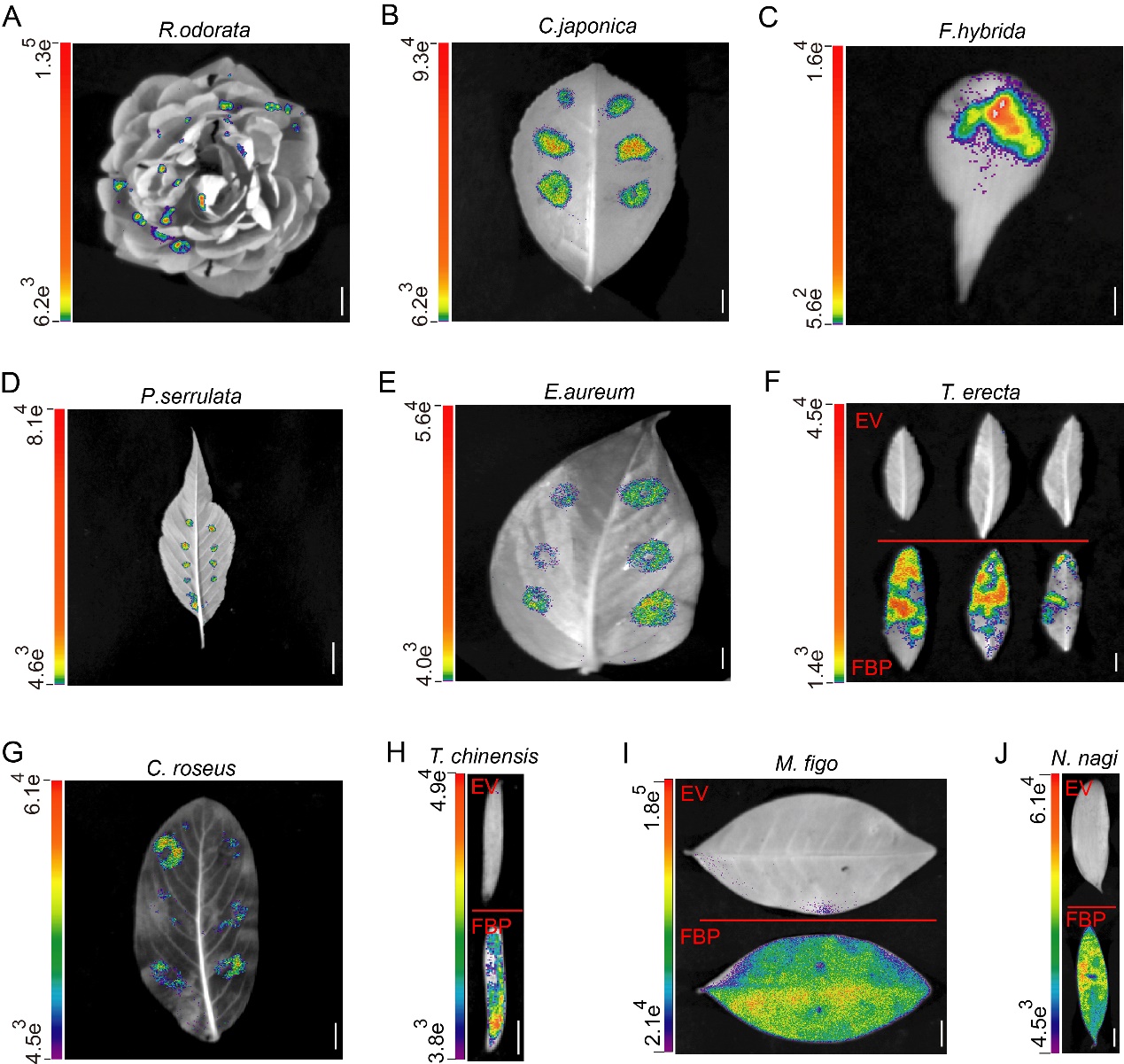


**Figure S8. The high-efficiency agroinfiltration of the tissues of multiple plant species.** (A-J) Optimized agroinfiltration of *Rosa odorata* petals (A), *Camellia japonica* leaves (B), *Freesia hybrida* petals (C), *Prunus serrulata* leaves (D), *Epipremnum aureum* leaves (E), *Tagetes erecta* leaves (F), *Catharanthus roseus* leaves (G), *Taxus chinensis* (H), *Michelia figo* leaves (I), *Nageia nagi* leaves (J) tissues with FBP and EV (empty vector), respectively. Bioluminescent image captured by photographic instrument from agroinfiltrated tissues after 72 h. Scale bars, 1 cm.

**Table S1** Candidate protein sequence used in this study.

**Table S2** Primers used in this study.

**Table S3** Vectors information used in this study.

**References**

Tamura, K., Stecher, G. and Kumar, S. (2021) MEGA11 Molecular Evolutionary Genetics Analysis Version 11. *Mol Biol Evol* **38**, 3022-3027.

Xi, Y.X., Chochois, V., Kroj, T. and Cesari, S. (2021) A novel robust and high-throughput method to measure cell death in leaves by fluorescence imaging. *Mol Plant Pathol* **22**, 1688-1696.
